# Sustainable nursing actions in health service waste management: a scoping review

**DOI:** 10.1590/0034-7167-2024-0027

**Published:** 2025-06-20

**Authors:** Ana Clara Dantas, Bárbara Ebilizarda Coutinho Borges, Anna Karla Regis de Azevedo, Mércio Gabriel de Araújo, Jéssica Naiara de Medeiros Araújo, Allyne Fortes Vitor

**Affiliations:** I Universidade Federal do Rio Grande do Norte. Natal, Rio Grande do Norte, Brazil; II Universidade do Estado do Rio Grande do Norte. Caicó, Rio Grande do Norte, Brazil; III Universidade Federal do Rio Grande do Norte. Santa Cruz, Rio Grande do Norte, Brazil

**Keywords:** Sustainable Development Indicators, Waste Management, Medical Waste, Nursing, Guidelines as Topic, Indicadores de Desarrollo Sostenible, Administración de Residuos, Residuos Sanitarios, Enfermería, Guías como Asunto

## Abstract

**Objective:**

to map evidence in the scientific literature about sustainable nursing actions in health service waste management.

**Methods:**

a scoping review, carried out in the Scopus, Web of Science, ScienceDirect, Medical Literature Analysis and Retrieval System Online (MEDLINE/PubMed), Embase, Google Scholar® databases and manual search in reference lists. The study was structured according to Preferred Reporting Items for Systematic Reviews and Meta-Analyses extension for Scoping Reviews recommendations.

**Results:**

the final sample consisted of 18 studies, most of which were published in 2023, in Europe, in English, with a quantitative approach and classified as level V of evidence.

**Conclusions:**

the sustainable nursing actions in waste management that most prevailed in the sample were related to material reduction, recycling and reuse. A diagram was created with the main sustainable nursing practices in health service waste management.

## RESUMEN

**Objetivo:**

mapear evidencia en la literatura científica sobre acciones sostenibles de enfermería en la gestión de resíduos de los servicios de salud.

**Métodos:**

revisión de alcance, realizada en bases de datos Scopus, Web of Science, ScienceDirect, Medical Literature Analysis and Retrieval System Online (MEDLINE/PubMed), Embase, Google Scholar® y búsqueda manual en listas de referencias. El estudio se estructuró de acuerdo con las recomendaciones del Preferred Reporting Items for Systematic Reviews and Meta-Analyses extension for Scoping Reviews.

**Resultados:**

la muestra final estuvo compuesta por 18 estudios, que en su mayoría fueron publicados en 2023, en el continente europeo, en idioma inglés, con enfoque cuantitativo y clasificados como nivel V de evidencia.

**Conclusiones:**

las acciones sostenibles de enfermería en la gestión de residuos que prevalecieron en la muestra estuvieron relacionadas con la reducción, el reciclaje y la reutilización de materiales. Se creó un diagrama con las principales prácticas sostenibles de enfermería en la gestión de residuos sanitarios.

## INTRODUCTION

In recent decades, definitions of sustainability have increasingly attracted researchers and professionals from around the world. Sustainability benefits current and future generations and refers to the balanced integration of three pillars: social, environmental and economic performance of life in society. This concept was proposed in order to support and operationalize the fulfillment of sustainable development^(1)^

In 2015, the United Nations (UN) proposed a set of 17 Sustainable Development Goals (SDGs) with the aim to “end poverty, protect the planet and improve the lives and prospects of everyone, everywhere” by 2030. The goals have an overview of 169 associated, integrated and indivisible targets, which include sustained economic growth, more sustainable consumption, production and resource use patterns, social development and environmental protection as fundamental pillars^(2)^.

To this end, in this final decade, government, organizational and community action was required to promote the implementation of local and global strategies to achieve the proposed goals. In this regard, nursing professionals were recognized as strong influencers in the approach to the SDGs, since they can cooperate positively in achieving health priorities through their workforce, global action and presence in remote areas, vulnerable populations and minority groups^(3)^.

Considering that the SDGs outline a global effort to protect the environment and its population, we can see the close relationship between nursing and SDG 12: “Ensure sustainable consumption and production patterns”. This SDG particularly has the proposed goal of significantly reducing waste generation through prevention, reduction, recycling and reuse, in addition to offering support to developing countries to consolidate their scientific and technological capabilities in promoting more sustainable consumption and production patterns, through research and guidelines that enable waste management^(2)^

Waste generated by healthcare services is classified as the second most dangerous waste in the world, and includes several types, such as sharp materials, fragments of human biological material, fluids, chemical and pharmaceutical waste, and medical devices. It comes from hospitals, health centers, doctors’ and dentists’ offices, veterinary clinics, pharmacies, among other healthcare services. International and national research shows concern about the amount of health waste produced, with the United States of America (USA) generating around 6 million tons and Brazil generating around 290 thousand tons per year^(4, 5)^.

There is an urgent need for healthcare professionals to rethink their practices in health service waste management as social agents so that it is possible to achieve lower levels of waste of inputs and waste production. From this perspective, nursing has introduced the environment as a decisive component in health promotion over time. Therefore, the responsible use of inputs and waste management in healthcare services are considered fundamental skills for training nursing professionals, as the sustainable use of instruments has an impact on the community’s socioeconomic aspect^(6)^.

However, there are obstacles in health waste management, such as inadequate understanding of management practices among healthcare professionals. This weakness can be explained by the lack of formal training, lack of knowledge about waste management and/or disposal in the workplace, and limited interest from those involved in the context^(7)^.

A recent study points to the need to develop research that considers environmentally sustainable actions in healthcare services, as well as information on good practices to minimize harmfulness and waste generation and maximize its recycling, in order to contribute to the environment and, consequently, to the health of current and future generations^(8)^.

SDG 12, in particular, has a focus on the management of waste produced in the context of healthcare, since this waste contributes to large-scale environmental pollution. Thus, gathering evidence on this topic in the field of nursing can favor the achievement of SDG 12 through the contribution of scientific knowledge, also relating to the achievement of SDG 3, which deals with ensuring healthy lives and promoting well-being for all^(9)^.

## OBJECTIVE

To map evidence in scientific literature about sustainable nursing actions in health service waste management.

## METHODS

### Ethical aspects

Since this was a study using secondary data from the public domain available in the literature, there was no need for ethical assessment. However, it is important to highlight that the copyright of the studies analyzed was duly respected, with correct citations and references.

### Study design

This is a scoping review, developed in accordance with the JBI Manual for Evidence Synthesis recommendations, prepared through the following stages: (1) research question elaboration; (2) relevant study identification; (3) study selection; (4) data mapping and analysis; and (5) synthesis of results^(10)^.

This study was guided by a research protocol previously developed and registered in the Open Science Framework (OSF) platform with the corresponding Uniform Resource Locator sequence identifier (osf.io/v3bwf)^(11)^. To guide the study report, the Preferred Reporting Items for Systematic Reviews and Meta-Analyses extension for Scoping Reviews (PRISMA-ScR) checklist was followed^(12)^.

### Research question development

The strategy used to develop the research question was based on the mnemonic PCC, in which P describes the population or research problem (nursing); C describes the concept (sustainable actions); and C describes the context (health service waste management). Thus, the following question was formulated: what are the sustainable nursing actions in health service waste management?

### Relevant study identification

Study identification occurred through three phases: database search; gray literature search; and manual search of reference lists. The first phase of the search was carried out through five databases, such as Scopus, Web of Science, ScienceDirect, Medical Literature Analysis and Retrieval System Online (MEDLINE/PubMed) and Embase. The last access date in the databases was November 6, 2023.

An advanced search was performed using descriptors indexed in the Medical Subject Headings (MeSH), such as “Guidelines as Topic”, “Waste Management”, “Medical Waste” and “Nursing”. To include a larger number of studies, alternative terms of the first descriptor were used, such as “Protocol” and “Normative Guidelines”. The Boolean operators “AND” and “OR” were used for cross-referencing in the search strategy, which met the particularities of each database.

The second phase of the search was carried out on Google Scholar®, using the initial descriptors and keywords identified in the studies from the first phase of the search. Thus, the keywords “Sustainable” and “Sustainable Actions” were added to the search strategy. The last access date on Google Scholar® was December 6, 2023. Chart 1 presents the strategies used in the data sources.

**Chart 1:** Search strategies used in each database, 2023

Database	Search strategy
Scopus	ALL(“Guidelines as Topic” OR Protocol OR “Normative guidelines”) AND (“Waste Management” OR “Medical Waste”) AND (Nursing)
Web of Science	((ALL=(“Guidelines as Topic” OR Protocol OR “Normative guidelines”)) AND ALL=(“Waste Management” OR “Medical Waste”)) AND ALL=(Nursing)
ScienceDirect	(“Guidelines as Topic” OR Protocol OR “Normative guidelines”) AND (“Waste Management” OR “Medical Waste”) AND (Nursing)
MEDLINE/PubMed	((“Guidelines as Topic” OR Protocol OR “Normative guidelines”) AND (“Waste Management” OR “Medical Waste”)) AND (Nursing)
Embase	(“Guidelines as Topic” OR Protocol OR “Normative guidelines”) AND (“Waste Management” OR “Medical Waste”) AND (Nursing)
Google Scholar®	“Guidelines as Topic” OR Protocol OR “Normative guidelines” AND “Waste Management” OR “Medical Waste” AND Nursing AND Sustainable OR “Sustainable Actions”

Studies that addressed the topic and were available in full were included. Editorials, letters to the editor, abstracts, correspondence and reviews were excluded from the sample. No limitations were imposed regarding the year of publication or language in order to include a larger number of studies.

### Study selection

The studies identified in data sources were exported to the Rayyan – Intelligent Systematic Review (https://rayyan.ai/), in which the study selection process took place by two researchers, independently, through dynamic reading of titles and abstracts of studies. The studies that met the eligibility criteria were classified as selected for full text reading, and those that did not meet the eligibility criteria were excluded from the sample. Selection discrepancies were resolved by a third researcher. Duplicate studies were counted in the sample only once.

It is worth noting that the second phase, carried out on Google Scholar®, was developed with the help of Publish or Perish (https://harzing.com/resources/publish-or-perish) to retrieve and analyze the identified results^(13)^. This made it possible to export the results of the second phase to Rayyan, in which the studies were selected.

The third phase of research was carried out by manually searching the reference lists of the studies selected in the previous phases. This process allowed the capture of studies that were not previously identified and that were considered relevant to the sample.

### Data mapping and analysis

For data mapping, an instrument was developed in Microsoft Excel 2019® with the following variables of interest: study identification data (title, indexed database, journal, country, authors, year of publication and language); methodological information of the study (study objective and/or research question, type of approach, study design and level of evidence (LoE)); waste management stage; category of health service waste; healthcare service environment; sustainable action description; nursing team role; and main conclusions.

In the LoE classification, the Polit and Beck (2021) framework was used, in which: level I: systematic review/meta-analysis of randomized controlled trials (RCTs); level II: RCTs; level III: non-randomized (quasi-experimental) trials; level IV: systematic review of non-experimental (observational) studies; level V: non-experimental/observational study; level VI: systematic review/meta-analysis of qualitative studies; level VII: qualitative/descriptive study; level VIII: source unrelated to the research (internal evidence and expert opinion)^(14)^.

During data analysis, a report bias assessment was performed. Studies that presented flaws in the report that could harm data analysis were removed from the sample to minimize bias.

### Summary of results

The results were summarized using figures, tables and charts to better visualize the data.

## RESULTS

**Figure 1:**
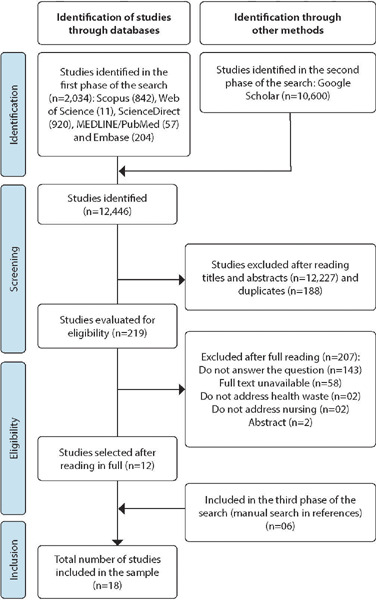
Flowchart of the study selection process, 2023

The search in data sources allowed identifying 12,634 studies, of which 188 were duplicates. After reading the titles and abstracts, 219 studies were assessed for eligibility. Of these, 207 were excluded after reading them in full. With the inclusion of the six studies through the manual search in reference lists, 18 studies comprised the final sample. Figure 1 shows the study selection process according to the PRISMA-ScR flowchart.

Study characterization was performed according to the year, continent of publication, language, type of approach, study design and LoE (Table 1).

**Table 1:** Characterization of studies according to year, continent of publication, language, type of approach, study design and level of evidence, 2023 (n=18)

Variables	n (%)
Year of publication	
2023	04 (22.2)
2022	02 (11.1)
2021	02 (11.1)
2020	02 (11.1)
2017	03 (16.6)
2015	01 (5.6)
2014	01 (5.6)
2013	02 (11.1)
2008	01 (5.6)
Continent	
Europe	08 (44.4)
South America	05 (27.7)
Asia	02 (11.1)
Africa	01 (5.6)
North America	01 (5.6)
Oceania	01 (5.6)
Language	
English	17 (94.4)
Portuguese	01 (5.6)
Type of approach	
Quantitative	12 (66.6)
Qualitative	03 (16.7)
Mixed	03 (16.7)
Study design	
Quasi-experimental study	03 (16.7)
Systematic review	03 (16.7)
Methodological study	03 (16.7)
Observational study	02 (11.1)
Qualitative study	02 (11.1)
Cross-sectional study	01 (5.5)
Descriptive study	01 (5.5)
Scoping review	01 (5.5)
Bibliometric analysis	01 (5.5)
Narrative review	01 (5.5)
Level of evidence	
Level V	06 (33.4)
Level VII	05 (27.7)
Level III	03 (16.7)
Level IV	03 (16.7)
Level VI	01 (5.5)

It is observed that 22.2% of studies were published in 2023, with 44.4% published in Europe and 94.4% published in English. Regarding the methodological aspects, 66.6% had a quantitative approach; quasi-experimental studies, systematic and methodological reviews prevailed in 16.7% in the study design variable; and 33.4% were classified as level V of evidence.

The summary of results with assigned code (ID), country, year of publication, study design, LoE, healthcare service and the main sustainable nursing actions in health service waste management is found in Chart 2.

**Chart 2:** Summary of results, 2023

ID	Country (year)	Study design/LoE*	Healthcare service	Sustainable nursing actions
A1^(15)^	Brazil (2023)	Scoping review/level IV	Hospital service	Reduce the amount of infectious and sharp waste and prefer disposable or single-use materials according to the lowest environmental impact.
A2^(16)^	USA (2023)	Systematic review/level VI	Hospital service	Reduce the stock of unnecessary supplies in hospital clothing/lab coat pockets.
A3^(17)^	Ukraine (2023)	Bibliometric analysis/level VII	Hospital service	Recycle/reuse plastics, paper, light bulbs, batteries and printer supplies.
A4^(18)^	Australia (2023)	Qualitative study/level VII	Hospital service	Use reusable bedding; conduct environmental education training; separate immunization boxes for recycling; implement a customized policy/Standard Operating Procedure (SOP) for sustainable actions; reduce the use of gloves in situations that do not require the use of Personal Protective Equipment (PPE).
A5^(19)^	Greece (2022)	Observational study/level V	Hospital service	Recycle/reuse plastics, paper, light bulbs, batteries and printer supplies; conduct environmental education training; implement a customized policy/SOP of sustainable actions.
A6^(20)^	South Korea (2022)	Descriptive study/level VII	Hospital service	Segregate common waste to improve health waste management; avoid routinely opening care products or surgical instrument kits.
A7^(21)^	United Kingdom (2021)	Systematic review/level IV	Hospital service	Segregate common waste to improve health waste management; recycle/reuse plastics, paper, light bulbs, batteries and printer supplies; use reusable containers for sharps; donate unused opened products; use reusable bed linen.
A8^(22)^	Egypt (2021)	Narrative review/level VII	Hospital service	Segregate common waste to improve health waste management; recycle/reuse plastics, paper, light bulbs, batteries and printer supplies; conduct environmental education training.
A9^(23)^	Greece (2020)	Methodological/level V	Hospital service	Recycle/reuse plastics, paper, light bulbs, batteries and printer supplies; conduct environmental education training; designate an environmental manager on the team; establish a common vision or values of sustainability.
A10^(24)^	Finland (2020)	Methodological study/level V	Hospital service	Prefer to administer oral medication in situations that do not require injections; avoid routinely opening care products or surgical instrument kits; reduce the use of gloves in situations that do not require the use of PPE; place orders for appropriate medications in the units, avoiding unnecessary expiration dates and waste.
A11^(25)^	Brazil (2017)	Quasi-experimental study/level III	Hospital service	Recycle/reuse plastics, paper, light bulbs, batteries and printer supplies; provide training on environmental education; reduce the amount of infectious and sharp waste.
A12^(26)^	India (2017)	Methodological study/level V	Hospital service	Segregate common waste to improve health waste management.
A13^(27)^	United Kingdom (2017)	Qualitative study/level VII	Hospital service	Segregate common waste to improve health waste management; provide continuous feedback on sustainable management.
A14^(28)^	Brazil (2015)	Quasi-experimental study/level III	Hospital service	Conduct training on environmental education; reduce excess packaging without compromising the organization of medications.
A15^(29)^	Brazil (2014)	Observational study/level V	Basic Health Unit	Conduct environmental education training; correctly identify storage containers.
A16^(30)^	Brazil (2013)	Quasi-experimental study/level III	Basic Health Unit	Recycle/reuse plastics, paper, light bulbs, batteries and printer supplies; conduct environmental education training; implement a customized policy/SOP of sustainable actions; promote educational campaigns to engage patients and visitors.
A17^(31)^	Finland (2013)	Systematic review/level IV	Hospital service	Recycle/reuse plastics, paper, light bulbs, batteries and printer supplies.
A18^(32)^	Greece (2008)	Cross-sectional study/level V	Hospital service	Segregate common waste to improve health waste management; recycle/reuse plastics, paper, light bulbs, batteries and printer supplies; conduct environmental education training.

Note: *LoE - level of evidence according to the Polit and Beck classification (2021) ^(14)^.

**Figure 2:**
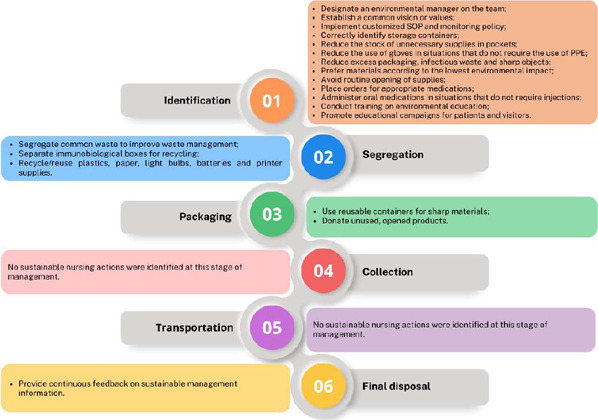
Diagram of health service waste management and sustainable nursing actions, 2023

It is evident that the main sustainable nursing actions in health service waste management were: recycling/reusing plastics, paper, light bulbs, batteries and printer supplies^(17, 19, 21, 23, 25, 30, 31, 32)^; carry out training on environmental education^(18, 19, 22, 23, 25, 28, 29, 30, 32)^; segregating common waste to improve health waste management^(20, 21, 22, 26, 27, 32)^; and implementing customized SOP and sustainable actions monitoring policy^(18, 19, 30)^. A diagram was developed to facilitate understanding regarding sustainable nursing actions during the health service waste management stages (Figure 2).

## DISCUSSION

When analyzing the studies identified in the scoping review, it was observed that the thematic area under study is an emerging research area, with little existing literature that specifically addresses sustainable practices in health service waste management. In general, sustainable nursing actions are related to improvements in waste reduction, recycling and sustainable reuse.

Considering the UN Global Plan of Action for Sustainable Development, sustainable actions should focus on reducing waste generation, understanding that reduction has a lower impact than recycling materials after disposal. Therefore, even though hospital waste management after disposal is extremely important, environmental management in healthcare services should also include sustainable practices that include reducing waste production and reusing resources^(28)^.

Supporting the findings of this study, Law 12.305/2010, which deals with Brazil’s National Solid Waste Policy (In Portuguese, *Política Nacional de Resíduos Sólidos* - PNRS), recommends the adoption of sustainable habits and instruments that favor the recycling and reuse of those inputs that have economic value and that provide opportunities for reuse as well as the environmentally effective disposal of those that cannot be reused^(33)^.

Among the main sustainable nursing actions in health service waste management identified, the recycling and reuse of materials such as plastics, paper, light bulbs, batteries and printer supplies stands out^(17, 19, 21, 23, 25, 30, 31, 32)^. The absence of a clear system for managing health waste, together with the lack of infrastructure to promote recycling, can result in unregulated disposal, causing soil, air and water pollution. However, many materials, such as plastics, paper and equipment, can be reused if appropriately segmented. It is observed that research tends to focus predominantly on waste collection and separation, with some neglect related to the recycling process^(17, 34)^.

Waste segregation emerges as a challenge in the adoption of sustainable practices, due to the lack of opportunity to segregate waste at the time of its generation in the healthcare environment^(20, 21, 22, 26, 27, 32)^. Some professionals point to the lack of adequate space or the absence of collectors that allow the storage of different types of waste, such as clinical or domestic waste. Thus, evidence suggests that the limitations may be associated with a lack of resources or an inadequate environment, and not necessarily a lack of willingness of professionals to change their waste management practices^(27, 35)^.

The studies addressed training on environmental education, recommending the implementation of online exhibitions on the topic at reception and in health units^(18, 19, 22, 23, 25, 28, 29, 30, 32)^. To this end, it is suggested that health institutions encourage and invest in formal education to improve professionals’ performance, especially in the context of environmental sustainability^(25)^. Confirming this finding, studies show that training and education programs in environmental sustainability significantly improve sustainable nursing practices^(36, 37)^.

Strategies such as implementing customized SOPs and policies for monitoring the quality and quantity of sustainable actions can improve environmental sustainability^(18, 19, 30)^. By visualizing indicators for monitoring sustainable practices, it becomes possible to provide continuous feedback in an assertive manner, supporting behavioral changes in relation to the sustainable management of health waste. Scientific literature also validates the use of feedback information as a means of driving behavioral transformations to encourage and sustain positive changes in sustainability management and promotion^(27, 30, 37)^.

In clinical practice, the use of disposable paper towels on hospital beds is observed, especially in outpatient consultations. However, studies indicate that the use of reusable bed linen is a more sustainable practice^(18, 21)^. Furthermore, opting for disposable or single-use materials, depending on the lowest environmental impact, is essential for sustainable practices^(15)^.

Nurses and other healthcare professionals often fail to make sufficient efforts to avoid waste and tend to use disposable products unnecessarily because they are not informed about sustainable practices. This includes the use of gloves in situations that do not require the use of PPE, such as when moving or feeding patients and administering vaccines. It is recommended that PPE materials be used only when necessary, in accordance with current regulations^(18, 24, 38)^. Furthermore, it is suggested to reduce the stock of unnecessary supplies in hospital clothing/lab coat pockets as a sustainable practice^(16)^.

Avoiding routinely opening care products or surgical instrument kits^(20, 24)^ and reducing the amount of infectious and sharps waste^(15, 25)^ have also been described in the studies. For instance, medications and medical supplies are purchased based on healthcare needs. Customized packages can be checked to identify unnecessary items, as they often contain products or instruments that are not used regularly. These supplies become health waste when they are used, discarded, or when their expiration date expires^(20)^.

It is common for expired medical supplies or those that have not been in contact with patients to be mistakenly disposed of as infectious medical waste or mixed with it for convenience, thus increasing the total volume of infectious medical waste generated. Therefore, reducing unnecessary infectious medical waste is feasible if healthcare professionals such as doctors, nurses and pharmacists are aware of the importance of correctly classifying waste^(20)^; place orders for medications with the appropriate quantity in the units, avoiding unnecessary expiration dates^(24)^; and make donations of unused open products for use in college teaching^(21)^.

Reducing excess packaging without compromising the organization of medications^(28)^, using reusable containers for sharps^(21)^, separating immunobiological boxes for recycling^(18)^ and administering oral medications in situations that do not require injections^(24)^ were also cited in the studies as sustainable nursing actions that improve waste management.

Another important factor for sustainable practices within the waste management system is the selection of professionals to guide sustainable healthcare services. For this purpose, designating an environmental manager represents an alternative for progress in institutions so that the team can work together in a committed manner to a more environmentally friendly healthcare service^(23, 39)^.

The consolidation of common values plays an important role in establishing a universal culture for all professionals with the aim of achieving collective goals. It is imperative to emphasize that sustainability promotion should not be based solely on positive individual behaviors, but should encompass environmental education at a collective level. In the specific field of nursing, the relevance of integrating these values is exemplified by studies that highlight the leadership role played by nurses in promoting environmental sustainability^(23, 37)^.

Nursing staff play an essential role in sustainable health education, providing detailed information on good practices related to health waste during educational campaigns, in order to engage patients and caregivers^(30, 40)^. This approach not only strengthens environmental sustainability in the healthcare sector, but also contributes to building an organizational culture that values and promotes socio-environmental responsibility.

The results of this study indicate that the entire nursing team can contribute consistently to the implementation of sustainable actions. To guide the team, it is recommended to develop clear and updated guidelines and protocols that involve sustainable actions in health service waste management.

Furthermore, it is suggested that health institutions promote and invest in educational programs to improve professionals’ performance, especially in the context of environmental sustainability. This can facilitate the integration of environmental sustainability objectives among nursing professionals, changes in attitudes and behaviors, in order to collaborate with the health of the planet and, consequently, with the health of people.

### Study limitations

The limitations of this study may be related to the eligibility criteria, which may have omitted research that addressed more innovative waste management strategies, due to the exclusion of studies on health service waste with professionals from other areas, such as dentistry or veterinary medicine.

### Contributions to nursing and public policies

This study contributes to the advancement of knowledge in nursing, aligning with the SDGs, especially SDGs 3 and 12, by highlighting sustainable actions in health service waste management based on the scoping review and by presenting a diagram of health service waste management and sustainable nursing actions.

For nursing professionals, these results have the potential to raise awareness about sustainable waste management, directly contributing to promoting sustainable consumption and production patterns, with a view to avoiding unnecessary waste and enabling a more efficient approach to resource use. A diagram development simplifies and visually clarifies waste management, making it more accessible for staff to adopt sustainable practices in their daily lives.

For managers, our study provides evidence-based information on practices that are both environmentally conscious and socially responsible. These practices can benefit the environment as well as positively impact the organizational culture of healthcare services. By having access to these results, managers can effectively promote more effective management of available resources, positively impacting climate change mitigation.

## CONCLUSIONS

The study mapped evidence about sustainable nursing actions in health service waste management, identifying methods that can reduce environmental impact and promote public health. The most prevalent actions found in the sample include material reduction, recycling and reuse, environmental education training and implementation of policies to monitor sustainable actions. Based on the findings of the review, a diagram was developed to help understand sustainable nursing actions at each stage of health service waste management. This resource can facilitate the adoption of sustainable practices by nursing professionals. The innovative nature of this study stands out for both nursing practice and the environment, since the implementation of identified practices can reduce the impact of health waste.
