# Supplementary material for: Sustainable nursing actions in health service waste management: a scoping review
Source: Rev Bras Enferm. 2025 Jun 20;78(Suppl 1):e20240027. doi: 10.1590/0034-7167-2024-0027 (PMC12185117; doi:10.1590/0034-7167-2024-0027)
Supplement: Translation

# Translation: Ações sustentáveis de enfermagem no gerenciamento de resíduos de serviços de saúde: revisão de escopo

**DOI:** 10.1590/0034-7167-2024-0027pt

**Authors:** Ana Clara Dantas, Bárbara Ebilizarda Coutinho Borges, Anna Karla Regis de Azevedo, Mércio Gabriel de Araújo, Jéssica Naiara de Medeiros Araújo, Allyne Fortes Vitor

**Affiliations:** I Universidade Federal do Rio Grande do Norte. Natal, Rio Grande do Norte, Brasil; II Universidade do Estado do Rio Grande do Norte. Caicó, Rio Grande do Norte, Brasil; III Universidade Federal do Rio Grande do Norte. Santa Cruz, Rio Grande do Norte, Brasil

**Keywords:** Indicadores de Desenvolvimento Sustentável, Gerenciamento de Resíduos, Resíduos de Serviços de Saúde, Enfermagem, Guias como Assunto

## Abstract

**Objetivo:**

mapear evidências na literatura científica acerca das ações sustentáveis de enfermagem no gerenciamento de resíduos de serviços de saúde.

**Métodos:**

revisão de escopo, realizada nas bases de dados Scopus, *Web of Science, Science Direct, Medical Literature Analysis and Retrieval System Online* (MEDLINE/PubMed), Embase, *Google Scholar*® e busca manual nas listas de referências. O estudo foi estruturado de acordo com as recomendações do *Preferred Reporting Items for Systematic Reviews and Meta-Analyses extension for Scoping Reviews.*

**Resultados:**

a amostra final foi composta por 18 estudos, que foram publicados em sua maioria em 2023, no continente europeu, no idioma inglês, com abordagem quantitativa e classificados com nível V de evidência.

**Conclusões:**

as ações sustentáveis de enfermagem no gerenciamento de resíduos que mais prevaleceram na amostra estavam relacionadas à redução, reciclagem e reutilização de materiais. Foi elaborado um diagrama com as principais práticas sustentáveis de enfermagem no gerenciamento de resíduos de serviços de saúde.

## INTRODUÇÃO

Nas últimas décadas, definições para sustentabilidade têm atraído progressivamente pesquisadores e profissionais de todo o mundo. A sustentabilidade beneficia gerações atuais e futuras, e se refere à integração de forma equilibrada de três pilares: desempenho social, ambiental e econômico da vida em sociedade. Essa conceituação foi proposta a fim de fundamentar e operacionalizar o cumprimento do desenvolvimento sustentável^(1)^.

Em 2015, foi proposto pela Organização das Nações Unidas (ONU) um conjunto de 17 Objetivos de Desenvolvimento Sustentável (ODS) com a perspectiva de “acabar com a pobreza, proteger o planeta e melhorar a vida e as perspectivas de todos, em todos os lugares” até o ano de 2030. Os objetivos possuem um panorama de 169 metas associadas, integradas e indivisíveis, as quais incluem o crescimento econômico sustentado, os padrões de consumo, de produção e de uso dos recursos mais sustentáveis, o desenvolvimento social e a proteção ambiental como pilares fundamentais^(2)^.

Para tanto, nesta década final, foram requeridas ações governamentais, organizacionais e de membros da comunidade em geral com vistas a favorecer a concretização de estratégias locais e globais para alcançar as metas propostas. Nesse sentido, os profissionais de enfermagem foram reconhecidos como fortes influentes na abordagem dos ODS, uma vez que podem cooperar positivamente no alcance das prioridades de saúde a partir de sua força de trabalho, atuação global e presença em áreas remotas, populações vulneráveis e grupos minoritários^(3)^.

Ao considerar que os ODS delineiam um esforço global com o propósito de proteger o ambiente e a sua população, observam-se a aproximação da enfermagem e o ODS 12: “Assegurar padrões sustentáveis de consumo e de produção”. Esta ODS particularmente possui a meta proposta de reduzir significativamente a geração de resíduos através da prevenção, redução, reciclagem e reutilização, além da oferta de suporte aos países em desenvolvimento para a consolidação de suas capacidades científicas e tecnológicas na promoção de padrões mais sustentáveis de consumo e produção, por meio de pesquisa e diretrizes que viabilizem o gerenciamento de resíduos^(2)^.

Os resíduos gerados pelos serviços de saúde são classificados como o segundo sedimento mais perigoso do mundo, e integram vários tipos, como materiais cortantes, fragmentos de materiais biológicos humanos, fluidos, resíduos químicos e farmacêuticos, e dispositivos médicos. São provenientes de hospitais, postos de saúde, consultórios médicos e odontológicos, clínicas veterinárias, farmácias, entre outros serviços de saúde. Pesquisas internacionais e nacionais demonstram preocupação com o quantitativo de resíduos de serviços de saúde produzidos, com os Estados Unidos da América (EUA) gerando cerca de 6 milhões de toneladas e o Brasil gerando cerca de 290 mil toneladas por ano^(4, 5)^.

Destaca-se a urgente necessidade de os profissionais de saúde repensarem suas práticas no gerenciamento de resíduos de serviços de saúde enquanto agentes sociais, de forma que seja possível alcançar níveis mais baixos de desperdício de insumos e produção de resíduos. Nessa perspectiva, a enfermagem introduziu ao longo do tempo o ambiente como componente decisivo na promoção da saúde. Por isso, o uso responsável de insumos e o gerenciamento de resíduos nos serviços de saúde são considerados habilidades fundamentais para a formação dos profissionais de enfermagem, na medida em que a utilização sustentável dos instrumentos repercute no aspecto socioeconómico da comunidade^(6)^.

No entanto, existem obstáculos no gerenciamento de resíduos de saúde, como o entendimento das práticas de gestão de maneira inapropriada entre os profissionais de saúde. Essa fragilidade pode ser explicada pela ausência de treinamento formal, privação de conhecimento acerca da administração dos resíduos e/ou descarte no ambiente de trabalho e o interesse limitado daqueles que estão inseridos no contexto^(7)^.

Estudo recente aponta a necessidade do desenvolvimento de pesquisas que considerem ações sustentáveis do ponto de vista ambiental nos serviços de saúde, bem como informações de boas práticas para minimizar a nocividade e a geração de resíduos e maximizar sua reciclagem, de modo a contribuir para o meio ambiente e, consequentemente, para a saúde das gerações atuais e futuras^(8)^.

O ODS 12, particularmente, possui uma vertente no gerenciamento de resíduos produzidos no contexto de saúde, visto que esses resíduos contribuem para a poluição ambiental em grande escala. Assim, o levantamento de evidências sobre essa temática no campo da enfermagem pode favorecer o alcance do ODS 12 através da contribuição do conhecimento científico, relacionan-do-se ainda com o alcance do ODS 3, que trata da asseguração de vidas saudáveis e promoção do bem-estar para todos^(9)^.

## OBJETIVO

Mapear evidências na literatura científica acerca das ações sustentáveis de enfermagem no gerenciamento de resíduos de serviços de saúde.

## MÉTODOS

### Aspectos éticos

Por se tratar de pesquisa com dados secundários de domínio público disponíveis na literatura, não houve a necessidade de apreciação ética. No entanto, é importante destacar que os direitos autorais dos estudos analisados foram devidamente respeitados, com citações e referências corretas.

### Desenho do estudo

Trata-se de revisão de escopo, desenvolvida de acordo com as recomendações do *JBI Manual for Evidence Synthesis,* elaborada por meio das seguintes etapas: (1) elaboração da questão de pesquisa; (2) identificação de estudos relevantes; (3) seleção dos estudos; (4) mapeamento e análise dos dados; e (5) síntese dos resultados^(10)^.

Este estudo foi orientado por um protocolo de pesquisa previamente desenvolvido e registrado na plataforma *Open Science Framework* (OSF) com o identificador sequencial correspondente de *Uniform Resource Locator* (osf.io/v3bwf)^(11)^. Para guiar o relatório do estudo, seguiu-se o *checklist Preferred Reporting Items for Systematic Reviews and Meta-Analyses extension for Scoping Reviews* (PRISMA-ScR)^(12)^.

### Elaboração da questão de pesquisa

A estratégia utilizada para elaboração da questão de pesquisa foi baseada no mnemônico PCC, em que P descreve a população ou problema da pesquisa (enfermagem); C descreve o conceito (ações sustentáveis); e C descreve o contexto (gerenciamento de resíduos de serviços de saúde). Desse modo, foi formulada a seguinte questão: quais as ações sustentáveis de enfermagem no gerenciamento de resíduos de serviços de saúde?

### Identificação de estudos relevantes

A identificação de estudos ocorreu mediante três fases: busca nas bases de dados; busca na literatura cinzenta; e busca manual nas listas de referências. A primeira fase da busca foi realizada por meio de cinco bases de dados, tais como Scopus, *Web of Science, ScienceDirect, Medical Literature Analysis and Retrieval System Online* (MEDLINE/PubMed) e Embase. A última data de acesso nas bases de dados foi em 06 de novembro de 2023.

Foi realizada uma busca avançada por intermédio de descritores indexados no *Medical Subject Headings* (MeSH), tais como “*Guidelines as Topic”, “Waste Management”, “Medical Waste”* e “*Nursing”.* Para englobar um maior quantitativo de estudos, utilizaram-se os termos alternativos do primeiro descritor, tais como “*Protocol”* e “*Normative Guidelines”.* Os operadores booleanos “*AND”* e “OR” foram utilizados para o cruzamento na estratégia de busca, que atendeu às particularidades de cada base de dados.

A segunda fase da busca foi realizada no *Google Scholar*®, com uso dos descritores iniciais e palavras-chave identificados nos estudos da primeira fase da busca. Dessa forma, as palavras-chave “*Sustainable” e “Sustainable Actions*” foram adicionadas na estratégia de busca. A última data de acesso no *Google Scholar*® foi 06 de dezembro de 2023. As estratégias utilizadas nas fontes de dados estão apresentadas no Quadro 1 a seguir.

**Quadro 1:** Estratégias de busca utilizada em cada base de dados, 2023

Base de dados	Estratégia de busca
Scopus	*ALL(“Guidelines as Topic” OR Protocol OR “Normative guidelines”) AND (“Waste Management” OR “Medical Waste”) AND (Nursing)*
*Web of Science*	*((ALL=(“Guidelines as Topic” OR Protocol OR “Normative guidelines”)) AND ALL=(“Waste Management” OR “Medical Waste”)) AND ALL=(Nursing)*
*Science Direct*	*(“Guidelines as Topic” OR Protocol OR “Normative guidelines”) AND (“Waste Management” OR “Medical Waste”) AND (Nursing)*
MEDLINE/PubMed	*((“Guidelines as Topic” OR Protocol OR “Normative guidelines”) AND (“Waste Management” OR “Medical Waste”)) AND (Nursing)*
Embase	*(“Guidelines as Topic” OR Protocol OR “Normative guidelines”) AND (“Waste Management” OR “Medical Waste”) AND (Nursing)*
*Google Scholar*®	*“Guidelines as Topic” OR Protocol OR “Normative guidelines” AND “Waste Management” OR “Medical Waste” AND Nursing AND Sustainable OR “Sustainable Actions”*

Incluíram-se estudos que abordassem a temática e que estivessem disponíveis na íntegra. Os editoriais, cartas ao editor, resumos, correspondências e resenhas foram excluídos da amostra. Não foram realizadas quaisquer limitações em relação ao ano de publicação e ao idioma com o intuito de abranger um número maior de estudos.

### Seleção dos estudos

Os estudos identificados nas fontes de dados foram exportados para o *software* Rayyan – *Intelligent Systematic Review* (https://rayyan.ai/), no qual ocorreu o processo de seleção dos estudos por dois pesquisadores, de forma independente, por meio da leitura dinâmica dos títulos e resumos dos estudos. Os estudos que atenderam aos critérios de elegibilidade foram classificados como selecionados para a leitura completa do texto, e os que não atenderam foram excluídos da amostra. As divergências de seleção foram resolvidas por um terceiro pesquisador. Os estudos duplicados foram contabilizados na amostra uma única vez.

Ressalta-se que a segunda fase, realizada no *Google Scholar*®, foi desenvolvida com o auxílio do *software Publish or Perish* (https://harzing.com/resources/publish-or-perish) para recuperar e analisar os resultados identificados^(13)^. Com isso, foi possível exportar os resultados da segunda fase para o *software* Rayyan, no qual procedeu-se à seleção dos estudos.

A terceira fase da busca foi realizada através da busca manual nas listas de referências dos estudos selecionados nas fases anteriores. Este processo permitiu a captação de estudos que não foram identificados previamente e que foram considerados relevantes para a amostra.

### Mapeamento e análise dos dados

Para o mapeamento dos dados, foi desenvolvido um instrumento no *Microsoft Excel 2019*® com as seguintes variáveis de interesse: dados de identificação do estudo (título, base de dados indexada, periódico, país, autores, ano de publicação e idioma); informações metodológicas do estudo (objetivo do estudo e/ou questão de pesquisa, tipo de abordagem, desenho do estudo e nível de evidência (NE)); etapa do gerenciamento de resíduos; categoria do resíduo de serviços de saúde; ambiente do serviço de saúde; descrição da ação sustentável; papel da equipe de enfermagem; e principais conclusões.

Na classificação do NE, utilizou-se o referencial de Polit e Beck (2021), no qual: nível I: revisão sistemática/meta-análise de ensaios controlados randomizados (ECR); nível II: ECR; nível III: ensaio não randomizado (quase-experimental); nível IV: revisão sistemática de estudos não experimentais (observacionais); nível V: estudo não experimental/observacional; nível VI: revisão sistemática/meta-análise de estudos qualitativos; nível VII: estudo qualitativo/descritivo; nível VIII: fonte não relacionada à pesquisa (evidência interna e opinião de especialistas)^(14)^.

Ainda durante a análise dos dados, foi realizada a avaliação de viés de relatório. Os estudos que apresentassem falhas no relatório que viessem a trazer prejuízos para análise dos dados foram removidos da amostra para minimizar vieses.

### Síntese dos resultados

A síntese dos resultados foi realizada por meio de figuras, tabelas e quadros, para melhor visualização dos dados.

## RESULTADOS

A busca nas fontes de dados possibilitou a identificação de 12.634 estudos, dos quais 188 eram duplicados. Após a leitura dos títulos e resumos, 219 estudos foram avaliados para elegibilidade. Destes, 207 foram excluídos após a leitura na íntegra. Com a inclusão dos seis estudos através da busca manual nas listas de referências, 18 estudos compuseram a amostra final. A Figura 1 apresenta o processo de seleção dos estudos de acordo com o fluxograma PRISMA-ScR.

**Figura 1:** Fluxograma do processo de seleção dos estudos, 2023

A caracterização dos estudos foi realizada de acordo com o ano, continente de publicação, idioma, tipo de abordagem, desenho do estudo e NE (Tabela 1).

**Tabela 1:** Caracterização dos estudos conforme ano, continente de publicação, idioma, tipo de abordagem, desenho do estudo e nível de evidência, 2023 (n=18)

Variáveis	n (%)
Ano de publicação	
2023	04 (22,2)
2022	02 (11,1)
2021	02 (11,1)
2020	02 (11,1)
2017	03 (16,6)
2015	01 (5,6)
2014	01 (5,6)
2013	02 (11,1)
2008	01 (5,6)
Continente	
Europa	08 (44,4)
América do Sul	05 (27,7)
Ásia	02 (11,1)
África	01 (5,6)
América do Norte	01 (5,6)
Oceania	01 (5,6)
Idioma	
Inglês	17 (94,4)
Português	01 (5,6)
Tipo de abordagem	
Quantitativo	12 (66,6)
Qualitativo	03 (16,7)
Misto	03 (16,7)
Desenho do estudo	
Estudo quase-experimental	03 (16,7)
Revisão sistemática	03 (16,7)
Estudo metodológico	03 (16,7)
Estudo observacional	02 (11,1)
Estudo qualitativo	02 (11,1)
Estudo transversal	01 (5,5)
Estudo descritivo	01 (5,5)
Revisão de escopo	01 (5,5)
Análise bibliométrica	01 (5,5)
Revisão narrativa	01 (5,5)
Nível de evidência	
Nível V	06 (33,4)
Nível VII	05 (27,7)
Nível III	03 (16,7)
Nível IV	03 (16,7)
Nível VI	01 (5,5)

Observa-se que 22,2% dos estudos foram publicados em 2023, com 44,4% publicados no continente europeu e 94,4% publicados no idioma inglês. Com relação aos aspectos metodológicos, 66,6% tinham abordagem quantitativa; os estudos quase-experimentais, revisão sistemática e metodológica prevaleceram em 16,7% na variável de desenho do estudo; e 33,4% foram classificados com nível V de evidência.

A síntese dos resultados com código atribuído (ID), país, ano de publicação, desenho do estudo, NE, serviço de saúde e as principais ações sustentáveis de enfermagem no gerenciamento de resíduos de serviços de saúde encontra-se no Quadro 2.

**Quadro 2:** Síntese dos resultados, 2023

ID	País (ano)	Desenho do estudo/NE*	Serviço de saúde	Ações sustentáveis de enfermagem
A1^(15)^	Brasil (2023)	Revisão de escopo/nível IV	Serviço hospitalar	Reduzir a quantidade de resíduos infectantes e perfurocortantes e preferir materiais descartáveis ou de uso único de acordo com o menor impacto ambiental.
A2^(16)^	Estados Unidos (2023)	Revisão sistemática/nível VI	Serviço hospitalar	Reduzir o estoque de suprimentos desnecessários em bolsos de roupa hospitalar/jaleco.
A3^(17)^	Ucrânia (2023)	Análise bibliométrica/nível VII	Serviço hospitalar	Reciclar/reutilizar plásticos, papéis, lâmpadas, baterias e insumos para impressoras.
A4^(18)^	Austrália (2023)	Estudo qualitativo/nível VII	Serviço hospitalar	Utilizar roupas de cama reutilizáveis; realizar treinamentos sobre educação ambiental; separar as caixas de imunização para reciclagem; implementar uma política/Procedimento Operacional Padrão (POP) customizado de ações sustentáveis; reduzir o uso de luvas em situações que não requerem o uso de Equipamento de Proteção Individual (EPI).
A5^(19)^	Grécia (2022)	Estudo observacional/nível V	Serviço hospitalar	Reciclar/reutilizar plásticos, papéis, lâmpadas, baterias e insumos para impressoras; realizar treinamentos sobre educação ambiental; implementar uma política/POP customizado de ações sustentáveis.
A6^(20)^	Coreia do Sul (2022)	Estudo descritivo/nível VII	Serviço hospitalar	Segregar o lixo comum para a melhoria da gestão dos resíduos de saúde; evitar abertura rotineira de produtos de cuidados ou *kits* de instrumentos cirúrgicos.
A7^(21)^	Reino Unido (2021)	Revisão sistemática/nível IV	Serviço hospitalar	Segregar o lixo comum para a melhoria da gestão dos resíduos de saúde; reciclar/reutilizar plásticos, papéis, lâmpadas, baterias e insumos para impressoras; utilizar recipientes reutilizáveis para materiais perfurocortantes; realizar doação de produtos abertos não utilizados; utilizar roupas de cama reutilizáveis.
A8^(22)^	Egito (2021)	Revisão narrativa/nível VII	Serviço hospitalar	Segregar o lixo comum para a melhoria da gestão dos resíduos de saúde; reciclar/reutilizar plásticos, papéis, lâmpadas, baterias e insumos para impressoras; realizar treinamentos sobre educação ambiental.
A9^(23)^	Grécia (2020)	Metodológico/nível V	Serviço hospitalar	Reciclar/reutilizar plásticos, papéis, lâmpadas, baterias e insumos para impressoras; realizar treinamentos sobre educação ambiental; designar um responsável ambiental na equipe; estabelecer uma visão ou valores comuns de sustentabilidade.
A10^(24)^	Finlândia (2020)	Estudo metodológico/nível V	Serviço hospitalar	Preferir administrar medicação oral em situações que não requerem injeções; evitar abertura rotineira de produtos de cuidados ou *kits* de instrumentos cirúrgicos; reduzir o uso de luvas em situações que não requerem o uso de EPI; fazer pedidos de medicamentos apropriados nas unidades, evitando vencimentos desnecessários e desperdício.
A11^(25)^	Brasil (2017)	Estudo quase-experimental/nível III	Serviço hospitalar	Reciclar/reutilizar plásticos, papéis, lâmpadas, baterias e insumos para impressoras; realizar treinamentos sobre educação ambiental; reduzir a quantidade de resíduos infectantes e perfurocortantes.
A12^(26)^	Índia (2017)	Estudo metodológico/nível V	Serviço hospitalar	Segregar o lixo comum para a melhoria da gestão dos resíduos de saúde.
A13^(27)^	Reino Unido (2017)	Estudo qualitativo/nível VII	Serviço hospitalar	Segregar o lixo comum para a melhoria da gestão dos resíduos de saúde; dar *feedback* contínuo de informações sobre o gerenciamento sustentável.
A14^(28)^	Brasil (2015)	Estudo quase-experimental/nível III	Serviço hospitalar	Realizar treinamentos sobre educação ambiental; reduzir excesso de embalagens sem comprometer a organização dos medicamentos.
A15^(29)^	Brasil (2014)	Estudo observacional/nível V	Unidade Básica de Saúde	Realizar treinamentos sobre educação ambiental; identificar corretamente recipientes de armazenamento.
A16^(30)^	Brasil (2013)	Estudo quase-experimental/nível III	Unidade Básica de Saúde	Reciclar/reutilizar plásticos, papéis, lâmpadas, baterias e insumos para impressoras; realizar treinamentos sobre educação ambiental; implementar uma política/POP customizado de ações sustentáveis; promover campanhas educativas para engajamento de pacientes e visitantes.
A17^(31)^	Finlândia (2013)	Revisão sistemática/nível IV	Serviço hospitalar	Reciclar/reutilizar plásticos, papéis, lâmpadas, baterias e insumos para impressoras.
A18^(32)^	Grécia (2008)	Estudo transversal/nível V	Serviço hospitalar	Segregar o lixo comum para a melhoria da gestão dos resíduos de saúde; reciclar/reutilizar plásticos, papéis, lâmpadas, baterias e insumos para impressoras; realizar treinamentos sobre educação ambiental.

Nota: *NE - nível de evidência de acordo com a classificação de Polit e Beck (2021) ^(14)^.

Evidencia-se que as principais ações sustentáveis de enfermagem no gerenciamento de resíduos de serviços de saúde foram: reciclar/reutilizar plásticos, papéis, lâmpadas, baterias e insumos para impressoras^(17, 19, 21, 23, 25, 30, 31, 32)^; realizar treinamentos sobre educação ambiental^(18, 19, 22, 23, 25, 28, 29, 30, 32)^; segregar o lixo comum para a melhoria da gestão dos resíduos de saúde^(20, 21, 22, 26, 27, 32)^; e implementar POP customizado e política de monitoramento de ações sustentáveis^(18, 19, 30)^. Foi desenvolvido um diagrama para facilitar a compreensão em relação às ações sustentáveis de enfermagem durante as etapas de gerenciamento de resíduos de serviços de saúde (Figura 2).

**Figura 2:** Diagrama do gerenciamento de resíduos de serviços de saúde e ações sustentáveis de enfermagem, 2023

## DISCUSSÃO

Ao analisar os estudos identificados na revisão de escopo, observou-se que a área da temática em estudo é uma área de pesquisa emergente, com pouca literatura existente que aborde especificamente práticas sustentáveis no gerenciamento de resíduos de serviços de saúde. De forma geral, as ações sustentáveis de enfermagem estão relacionadas às melhorias na redução, reciclagem e reutilização sustentável de resíduos.

Considerando o Plano de Ação Global para o alcance do Desenvolvimento Sustentável da ONU, as ações sustentáveis devem dar enfoque na redução de geração de resíduos, ao compreender que a redução causa menor impacto do que reciclar os materiais após o seu descarte. Portanto, mesmo que o manejo de resíduos hospitalares após descarte seja de extrema importância, a gestão ambiental nos serviços de saúde deve abranger também práticas sustentáveis que incluam a redução da produção de resíduos e o reaproveitamento de recursos^(28)^.

Corroborando os achados deste estudo, a Lei nº 12.305/2010, que trata da Política Nacional de Resíduos Sólidos (PNRS) do Brasil, recomenda a adoção de hábitos sustentáveis e de instrumentos que favoreçam a reciclagem e a reutilização daqueles insumos que detêm valor económico e que oportunizem o reaproveitamento, bem como a destinação ambientalmente efetiva para aqueles que não são capazes de serem reutilizados^(33)^.

Entre as principais ações sustentáveis de enfermagem no gerenciamento de resíduos de serviços de saúde identificadas, destaca-se a reciclagem e reutilização de materiais como plásticos, papéis, lâmpadas, baterias e insumos para impressoras^(17, 19, 21, 23, 25, 30, 31, 32)^. A ausência de um sistema claro para a gestão dos resíduos de saúde, juntamente com a falta de infraestrutura para promover a reciclagem, pode resultar na eliminação não regulamentada, causando poluição do solo, ar e água. No entanto, é possível reutilizar muitos materiais, como plásticos, papéis e equipamentos, mediante uma segmentação adequada. Observa-se que as pesquisas tendem a enfocar predominantemente a coleta e a separação dos resíduos, com certa negligência relacionada ao processo de reciclagem^(17, 34)^.

A segregação dos resíduos surge como um desafio na adoção de práticas sustentáveis, devido à falta de oportunidade para segregá-los no momento de sua geração no ambiente de saúde^(20, 21, 22, 26, 27, 32)^. Alguns profissionais apontam a falta de espaço adequado ou a ausência de coletores que viabilizem o armazenamento de diferentes tipos de resíduos, como os de natureza clínica ou doméstica. Dessa forma, evidências sugerem que as limitações podem estar associadas à falta de recursos ou a um ambiente inadequado, e não necessariamente à falta de vontade dos profissionais em modificar suas ações na gestão de resíduos^(27, 35)^.

Os estudos abordaram treinamentos sobre educação ambiental, recomendando a implementação de exposições *online* sobre o tema na recepção e nas unidades de saúde^(18, 19, 22, 23, 25, 28, 29, 30, 32)^. Neste intuito, sugere-se que as instituições de saúde incentivem e invistam na educação formal para aprimorar o desempenho dos profissionais, especialmente no contexto da sustentabilidade ambiental^(25)^. Confirmando esse achado, estudos apontam que os treinamentos e programas de formação em sustentabilidade ambiental melhoram significativamente as práticas sustentáveis de enfermagem^(36, 37)^.

Estratégias como a implementação de POP customizados e políticas de monitoramento de qualidade e quantidade de ações sustentáveis podem melhorar a sustentabilidade ambiental^(18, 19, 30)^. Ao visualizar indicadores de monitoramento das práticas sustentáveis, torna-se possível fornecer *feedback* contínuo de maneira assertiva, apoiando mudanças de comportamento em relação à gestão sustentável de resíduos de saúde. A literatura científica também valida o uso de informações em *feedback* como meio de impulsionar transformações comportamentais para estimular e sustentar mudanças positivas na gestão e promoção da sustentabilidade^(27, 30, 37)^.

Na prática clínica, observa-se o uso de toalhas de papéis descartáveis em camas hospitalares, principalmente em consultas ambulatoriais. No entanto, estudos indicam que a utilização de roupas de cama reutilizáveis é uma prática mais sustentável^(18, 21)^. Além disso, optar por materiais descartáveis ou de uso único, de acordo com o menor impacto ambiental, é essencial para as práticas sustentáveis^(15)^.

A enfermagem, frequentemente, e outros profissionais de saúde podem não fazer esforços suficientes para evitar desperdícios, e tendem a usar produtos descartáveis desnecessariamente por não estarem informados sobre as práticas sustentáveis. Isso inclui o uso de luvas em situações que não requerem o uso de EPI, como na mobilização ou alimentação de pacientes e na administração de vacinas. Orienta-se a utilização de materiais de EPI apenas quando forem necessários, conforme as normas vigentes^(18, 24, 38)^. Ainda, sugere-se a redução do estoque de suprimentos desnecessários em bolsos de roupa hospitalar/jaleco como uma prática sustentável^(16)^.

Evitar abrir rotineiramente produtos de cuidados ou *kits* de instrumentos cirúrgicos^(20, 24)^ e reduzir a quantidade de resíduos infectantes e perfurocortantes^(15, 25)^ igualmente foram descritos nos estudos. Por exemplo, medicamentos e suprimentos médicos são adquiridos com base nas necessidades dos serviços de saúde. Os pacotes personalizados podem ser verificados para identificar itens desnecessários, pois muitas vezes contêm produtos ou instrumentos que não são usados regularmente. Esses insumos se tornam resíduos de serviços de saúde quando são usados, descartados ou quando seu prazo de validade expira^(20)^.

É comum que suprimentos médicos vencidos ou que não estiveram em contato com os pacientes sejam erroneamente descartados como resíduos médicos infecciosos ou misturados a eles por conveniência, aumentando assim o volume total de resíduos médicos infecciosos gerados. Portanto, a redução de resíduos médicos infecciosos desnecessários é viável caso os profissionais de saúde, como médicos, enfermeiros e farmacêuticos, estejam cientes da importância de classificar corretamente os resíduos^(20)^; façam pedidos de medicamentos com a quantidade apropriada nas unidades, evitando vencimentos desnecessários^(24)^; e realizem doações de produtos abertos não utilizados para o uso no ensino em faculdades^(21)^.

Reduzir o excesso de embalagens sem comprometer a organização dos medicamentos^(28)^, utilizar recipientes reutilizáveis para materiais perfurocortantes^(21)^, separar as caixas de imunobiológicos para reciclagem^(18)^ e administrar medicamentos orais em situações que não requerem injeções^(24)^ também foram citadas nos estudos como ações sustentáveis de enfermagem que melhoram a gestão de resíduos.

Outro fator importante para as práticas sustentáveis dentro do sistema de gestão de resíduos é a escolha de profissionais para orientar serviços de saúde sustentáveis. Com esse propósito, designar um responsável ambiental representa uma alternativa de progresso das instituições para que a equipe possa compactuar de forma empenhada em um serviço de saúde mais ecológico^(23, 39)^.

A consolidação de valores em comum desempenha um papel importante na instauração de uma cultura universal a todos os profissionais com a finalidade de alcançar metas coletivas. É imperativo ressaltar que a promoção da sustentabilidade não deve ser firmada apenas em comportamentos individuais positivos, mas abranger a formação ambiental a nível coletivo. No campo específico da enfermagem, a relevância da integração desses valores é exemplificada por estudos que evidenciam o papel de liderança desempenhado pelos enfermeiros na promoção da sustentabilidade ambiental^(23, 37)^.

A equipe de enfermagem possui um papel essencial na educação em saúde sustentável, fornecendo informações detalhadas sobre boas práticas relacionadas aos resíduos de saúde durante campanhas educativas, a fim de engajar pacientes e acompanhantes^(30, 40)^. Essa abordagem não apenas fortalece a sustentabilidade ambiental na área da saúde, mas também contribui para a construção de uma cultura organizacional que valoriza e promove a responsabilidade socioambiental.

Os resultados deste estudo apontam que toda a equipe de enfermagem pode contribuir de maneira consistente para a implementação de ações sustentáveis. Para direcionar a equipe, recomenda-se a elaboração de diretrizes e protocolos claros e atualizados que envolvam ações sustentáveis no gerenciamento de resíduos de serviços de saúde.

Além disso, sugere-se que as instituições de saúde promovam e invistam em programas educacionais para aprimorar o desempenho dos profissionais, especialmente no contexto da sustentabilidade ambiental. Isso pode facilitar a integração dos objetivos de sustentabilidade ambiental aos profissionais de enfermagem, as mudanças de atitudes e comportamentos, de modo a colaborar com a saúde do planeta e, por conseguinte, com a saúde das pessoas.

### Limitações do estudo

As limitações deste estudo podem estar relacionadas aos critérios de elegibilidade, que podem ter omitido pesquisas que abordassem estratégias mais inovadoras de gerenciamento de resíduos, em razão da exclusão de estudos sobre resíduos de serviços de saúde com profissionais de outras áreas, como odontologia ou medicina veterinária.

### Contribuições para as áreas da enfermagem e políticas públicas

Este estudo contribui para o avanço do conhecimento na área da enfermagem, alinhando-se aos ODS, especialmente aos ODS 3 e 12, ao destacar ações sustentáveis no gerenciamento de resíduos de serviços de saúde a partir da revisão de escopo e ao apresentar um diagrama do gerenciamento de resíduos de serviços de saúde e ações sustentáveis de enfermagem.

Para os profissionais de enfermagem, esses resultados possuem potencial de conscientizar sobre a gestão sustentável de resíduos, contribuindo diretamente para promover padrões sustentáveis de consumo e de produção, com vistas a evitar desperdícios desnecessários e viabilizar uma abordagem mais eficiente no uso de recursos. O desenvolvimento do diagrama simplifica e esclarece de forma visual o gerenciamento de resíduos, tornando-o mais acessível para a equipe adotar práticas sustentáveis no dia a dia.

Para os gestores, nosso estudo fornece informações baseadas em evidências sobre práticas que são tanto ambientalmente conscientes quanto socialmente responsáveis. Essas práticas podem beneficiar o meio ambiente, bem como impactar de forma positiva a cultura organizacional dos serviços de saúde. Ao ter acesso a esses resultados, os gestores podem promover efetivamente um gerenciamento mais eficaz dos recursos disponíveis, impactando positivamente a mitigação das mudanças climáticas.

## CONCLUSÕES

O estudo mapeou evidências acerca das ações sustentáveis de enfermagem no gerenciamento de resíduos de serviços de saúde, identificando métodos que podem reduzir o impacto ambiental e promover a saúde pública. As ações mais prevalentes encontradas na amostra incluem a redução, reciclagem e reutilização de materiais, treinamentos sobre educação ambiental e implementação de políticas de monitoramento de ações sustentáveis.

Com base nos achados da revisão, foi elaborado um diagrama que ajuda a compreender as ações sustentáveis de enfermagem em cada etapa do gerenciamento de resíduos de serviços de saúde. Este recurso pode facilitar a adoção de práticas sustentáveis pelos profissionais de enfermagem. Destaca-se o caráter inovador deste estudo tanto para a prática da enfermagem quanto para o meio ambiente, uma vez que a implementação das práticas identificadas pode reduzir o impacto dos resíduos de saúde.
